# PKC and AKT Modulate cGMP/PKG Signaling Pathway on Platelet Aggregation in Experimental Sepsis

**DOI:** 10.1371/journal.pone.0137901

**Published:** 2015-09-16

**Authors:** M. Elisa Lopes-Pires, Ana C. Antunes Naime, Nádia J. Almeida Cardelli, Débora J. Anjos, Edson Antunes, Sisi Marcondes

**Affiliations:** Department of Pharmacology, Faculty of Medical Sciences, University of Campinas (UNICAMP), Campinas (SP), Brazil; Royal College of Surgeons, IRELAND

## Abstract

Sepsis severity has been positively correlated with platelet dysfunction, which may be due to elevations in nitric oxide (NO) and cGMP levels. Protein kinase C, Src kinases, PI3K and AKT modulate platelet activity in physiological conditions, but no studies evaluated the role of these enzymes in platelet aggregation in sepsis. In the present study we tested the hypothesis that in sepsis these enzymes positively modulate upstream the NO-cGMP pathway resulting in platelet inhibition. Rats were injected with lipopolysaccharide (LPS, 1 mg/kg, i.p.) and blood was collected after 6 h. Platelet aggregation was induced by ADP (10 *μ*M). Western blotting assays were carried out to analyze c-Src and AKT activation in platelets. Intraplatelet cGMP levels were determined by enzyme immunoassay kit. Phosphorylation of c-SRC at Tyr416 was the same magnitude in platelets of control and LPS group. Incubation of the non-selective Src inhibitor PP2 (10 μM) had no effect on platelet aggregation of LPS-treated rats. LPS increased intraplatelet cGMP levels by 5-fold compared with control group, which was accompanied by 76% of reduction in ADP-induced platelet aggregation. The guanylyl cyclase inhibitor ODQ (25 μM) and the PKG inhibitor Rp-8-Br-PET-cGMPS (25 μM) fully reversed the inhibitory effect of LPS on platelet aggregation. Likewise, the PKC inhibitor GF109203X (10 μM) reversed the inhibition by LPS of platelet aggregation and decreased cGMP levels in platelets. AKT phosphorylation at Thr308 was significantly higher in platelets of LPS compared with control group, which was not reduced by PI3K inhibition. The AKT inhibitor API-1 (20 μM) significantly increased aggregation and reduced cGMP levels in platelets of LPS group. However, the PI3K inhibitor wortmannin and LY29004 had no effect on platelet aggregation of LPS-treated rats. Therefore, inhibition of ADP-induced platelet aggregation after LPS injection is mediated by cGMP/PKG-dependent mechanisms, and PKC and AKT act upstream upregulating this pathway.

## Introduction

Platelets are important blood elements responsible for homeostasis maintenance and formation of pathological thrombus. Platelets can be activated by different agonists, including ADP that activates P2Y1 and P2Y12 receptors coupled to Gq and Gi proteins, respectively [1]. Stimulation of P2Y1 receptor leads to increase in cytosolic Ca^++^ concentration and PKC activation [2]. PKC in turn increases platelet secretion [3,4] and activates fibrinogen receptor (integrin α_IIb_β_3_) that mediates the outside-in signaling, triggering a series of intracellular events that lead to platelet spreading, stabilization of platelet aggregate and cytoskeletal reorganization [5,6]. On the other hand, stimulation of P2Y12 receptors activates PI3K, which is important for sustained α_IIb_β_3_ integrin activation [2,7]. Besides PI3K, other enzymes take part in the outside-in signaling, including c-Src, a member of Src family kinases (SFKs), which is bound to the cytoplasmic domain of the β_3_ integrin subunit [8,9]. Platelet activation can be inhibited by different mechanisms, including nitric oxide (NO) synthesis. Most of the effects of NO in platelets are mediated by activation of soluble guanylyl cyclase (sGC) and cGMP formation, which activates cGMP-dependent protein kinase (PKG) leading to inhibition of platelet aggregation through phosphorylation of different targets [10,11].

Studies showing the involvement of platelets in sepsis have been growing over the past few years. A positive correlation between platelet dysfunction and sepsis severity has been described [12–15]. Previous studies have shown that patients with sepsis exhibit reduced platelet aggregation to ADP, collagen and arachidonic acid [14,16]. Lipopolysaccharide (LPS) is postulated to play an important role in sepsis syndrome, and is frequently used to induce experimental sepsis. Similarly to septic patients, platelet aggregation is decreased in rats after intraperitoneal or intravenous LPS administration [17–19].

Despite of works showing the inhibitory effects of LPS on platelets, the intracellular mechanisms have not yet been elucidated. In the present work, we have investigated the role of SFKs, PI3K, AKT and PKC and sGC on platelet aggregation inhibition in experimental sepsis using specific inhibitors of these enzymes. Immunoblotting assays to determine the activation of c-Src and AKT, as well as measurements of intraplatelet cGMP levels were also performed.

## Material and Methods

### Reagents

Lipopolysaccharide from Escherichia coli (type 0111:B4), adenosine diphosphate (ADP), 2’,7’-dichlorofluorescein diacetate (DCFH-DA), wortmannin, 4-Amino-5-(4-chlorophenyl)-7-(t-butyl)pyrazolo[3,4-d]pyrimidine (PP2); bisindolilmaleimida I (GF 109203X), 1H-[1,2,4]oxadiazolo[4,3,-a]quinoxalin-1-one (ODQ), LY294002 and 3-isobutyl-l-methyl-xanthine (IBMX) were purchased from Sigma Chem. Co. (St. Louis, MO, USA). 4-Amino-5,8-dihydro-5-oxo-8-β-D-ribofuranosyl-pyrido[2,3-d]pyrimidine-6-carboxamide (API-1) and 2-Bromo-3,4-dihydro-3-[3,5-O-[(R)-mercaptophosphinylidene]-β-D-ribofuranosyl]-6-phenyl-9H-Imidazo[1,2-a]purin-9-one (Rp-8-Br-PET-cGMPS) were purchased from Tocris Bioscience House (Bristol, UK). Mouse monoclonal anti-cSrc, anti-cSrc phosho-Tyr416, anti-AKT1/PKBα and anti-AKT1/PKBα phospho-Thr308 antibodies were purchased from Millipore (Billerica, MA, USA). Horseradish peroxidase-conjugated secondary antibody was purchased from GE Healthcare Life Sciences (St Giles, Buckinghamshire, UK).

### Experimental protocols

All animal procedures and experimental protocols are in accordance with the Ethical Principles in Animal Research adopted by the Brazilian College for Animal Experimentation (COBEA) and were approved by the institutional Committee for Ethics in Animal Research/State University of Campinas (CEEA-UNICAMP, protocol 2097–1). Male Wistar rats (250–320 g) were housed in temperature-controlled rooms and received water and food ad libitum. Rats were treated with a single intraperitoneal (i.p) injection of saline (300 μl) or LPS (1 mg/kg). At 6 h thereafter the animals were anaesthetized with isoflurane, and blood was collected from abdominal aorta [19]. Washed platelet samples were separated to three independent experimental procedures, that is, (i) platelet aggregation in response to ADP, (ii) measurement of cGMP levels in IBMX-treated platelets and (iii) western blotting analysis for non-phosphorylated and phosphorylated Src, and non-phosphorylated and phosphorylated AKT. These experimental protocols are detailed below.

### Washed platelet preparation

Arterial blood was collected in 1:9 (v/v) of ACD-C (12.4 mM sodium citrate, 13 mM citric acid, 11 mM glucose). First, platelet-rich plasma (PRP) was obtained by centrifugation of whole blood at 200 g for 15 min at room temperature. Five milliliters of PRP were added to 7 ml of washing buffer (140 mM NaCl, 0.5 mM KCl, 12 mM trisodium citrate, 10 mM glucose, 12.5 mM saccharose, pH 6), and centrifuged (800 g, 13 min). The pellet was resuspended in washing buffer, and the procedure was repeated once. The platelets were suspended in Krebs solution (118 mM NaCl, 25 mM NaHCO_3_, 1.2 mM KH_2_PO_4_, 1.7 mM MgSO_4_, 5.6 mM glucose, pH 7.4). The platelet number was adjusted to 1.2 x 10^8^ platelets/ml in the presence of 1 mM CaCl_2_.

### Platelet aggregation

Platelet aggregation was measured in a two channel aggregometer (Chronolog Lumi-Aggregometer model 560-Ca, Havertown, PA, USA) at 37°C with stirring (1 000 rpm). Platelet aggregation assays were carried out using ADP (10 μM) and in some experiments the platelets were pre-incubated for 3 min with inhibitors of SFKs (PP2, 10 μM), PI3K (wortmannin, 100 nM; and LY294002, 10 μM), AKT (API-1, 20 μM), sGC (ODQ, 25 μM), PKG (Rp-8-Br-PET-cGMPS, 25 μM) or PKC (GF109203X, 10 μM). The concentrations of these inhibitors were used accordingly to previously published studies [20–24]. The same volume of DMSO (1%) was used as vehicle control for all the inhibitors, except for Rp-8-Br-PET-cGMPS that was dissolved in saline. Platelet aggregation data were obtained at an end-point of 5 min after ADP addition.

### Extraction and Measurement of cGMP from Platelets

Platelets (1.2 x 10^8^ platelets/ml) were incubated with the phosphodiesterase inhibitor IBMX (2 mM) for 15 min. Non-activated or ADP (10 μM)-activated platelets were incubated with PKC inhibitor GF109203X (10 μM), AKT inhibitor API-1 (20 μM) or the same volume of DMSO (0.1%) for 15 min. The reaction was interrupted by the addition of cold acidified absolute ethanol (67%, v/v), and samples were vigorously agitated for 30 s. Cell samples were centrifuged at 4,000 *g* (30 min, 4°C). Supernatants were dried at 55–60°C under a stream of nitrogen. Cyclic GMP was measured using a kit from Cayman Chemical Co. (Ann Arbor, MI).

### Western Blotting

Washed platelets (1.2 x 10^8^ platelets/ml) from saline- or LPS-injected rats were stimulated or not with ADP (10 μM) for 5 min with stirring. In some experiments, platelets were pre-incubated with the Src inhibitor PP2 (10 μM) or PI3K inhibitor LY29004 (10 μM) for 3 min before ADP addition. The platelet suspension was sonicated for 30 sec and centrifuged at 10,000 g for 10 min at 4°C. Protein concentrations of the supernatants were determined by the Lowry assay, and an equal amount of protein from each sample (25 μg or 50 μg) was treated with Laemmli buffer. Samples were resolved by SDS-PAGE (10%). After separation, proteins were electrophoretically transferred to PVDF membranes (90 min, 100 mA) and blocked for 30 min at room temperature in solution containing 1% of BSA in Tris-buffered saline-tween (TBS-T, 20 mM Tris-HCl pH7.2, 0.3 M NaCl with 0.1% Tween-20). The membrane was incubated overnight in blocking buffer containing mouse monoclonal anti-cSrc or anti-AKT (1:2000, Millipore, Billerica, MA, USA) at 4°C. After washing with TBS-T, immunoreactive proteins were detected by using horseradish peroxidase-conjugated secondary antibodies (GE Healthcare Life Sciences, St Giles, Buckinghamshire, UK) and enhanced chemiluminescence. Membranes were stripped and re-probed for anti-cSrc phospho-Tyr416 and anti-AKT phospho-Thr308 antibodies (1:2000, Millipore, Billerica, MA, USA). Densitometry was performed using the UN-SCAN-IT-gel 6.1 Software. The levels of phosphorylated c-Src or AKT were normalized to the total c-Src or AKT, respectively.

### Statistical analysis

Data are expressed as means ± SEM of *n* animals. The statistical significance between groups was determined by using ANOVA followed by the Tukey test. A *p* value of less than 0.05 was considered statistically significant.

## Results

### Effect of Src-family kinases (SFKs) inhibition on washed platelet aggregation of LPS-treated rats

**Fig 1:**
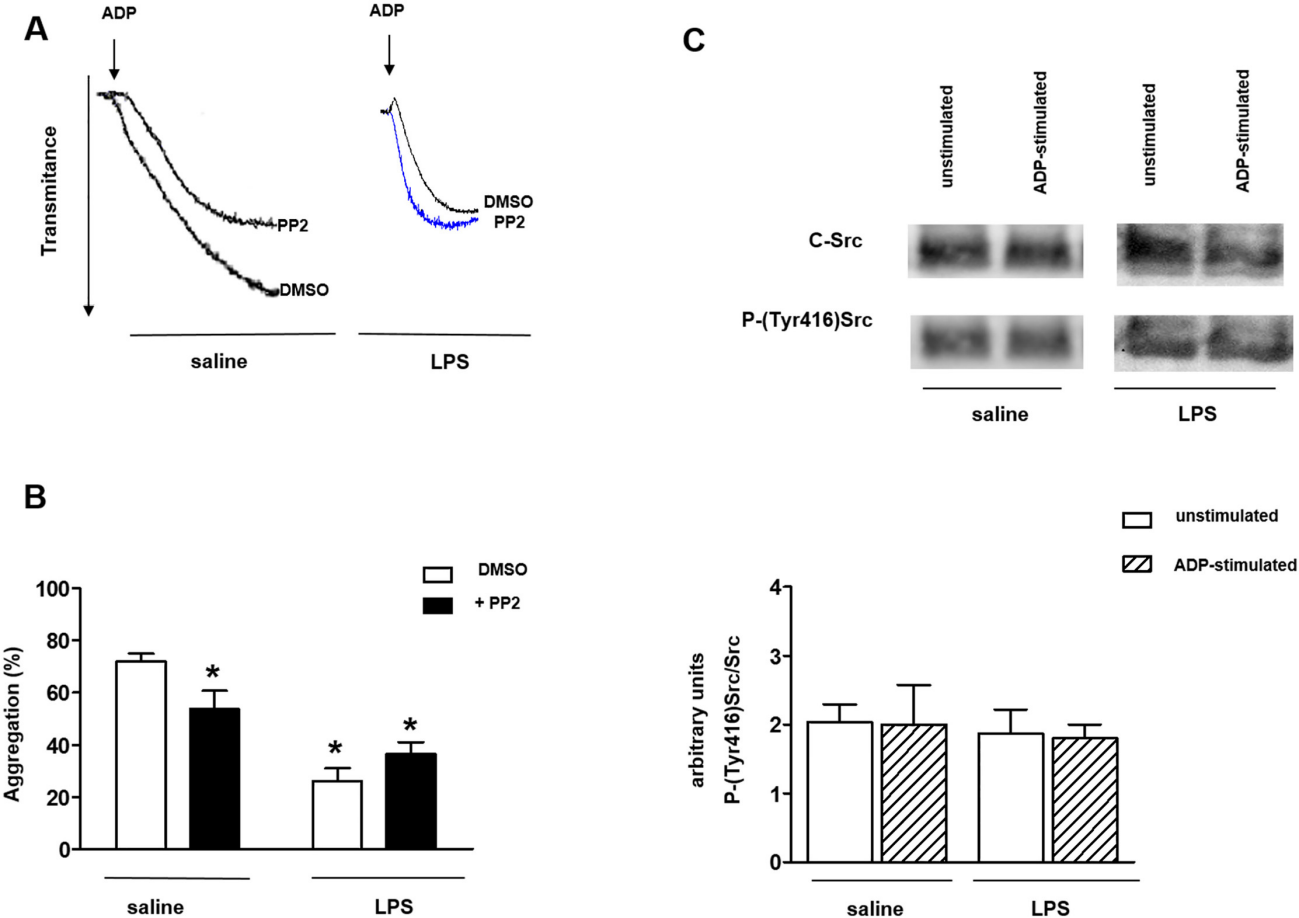
Inhibition of SFKs does not affect ADP-induced platelet aggregation of LPS-treated rats. Rats were injected with LPS (1 mg/kg) and after 6 h blood was collected. (Panels A and B) Washed platelets (1.2 x 10^8^ platelets/ml) were incubated with the SFK inhibitor PP2 (10 μM) or its vehicle (1% DMSO) for 3 min before addition of ADP (10 μM). (Panel C) Samples (25 μg of protein/well) were loaded on SDS-PAGE 10% and analyzed by Western blot using anti-cSrc or anti-phospho(Tyr 416)-Src antibodies. Graph shows densitometric analysis of immunoreactive bands. Results are shown as mean ± SEM values (*n* = 3–4 different animals). *P<0.05 compared with DMSO values in saline group.

Addition of ADP (10 μM) to washed platelet suspension (1.2 x10^8^ platelets/ml) of saline-injected rats induced a significant aggregation (Fig 1A and 1B, S1 Table). In vivo pre-treatment with LPS (1 mg/kg, i.p.) caused a significant reduction of ADP-induced platelet aggregation at 6 h post-LPS administration (Fig 1A and 1B, S1 Table). In saline-injected rats, incubation of platelets with the SFKs inhibitor PP2 (10μM, 3 min) significantly reduced the ADP-induced platelet aggregation (P<0.05). However, in LPS-treated rats, incubation of platelets with PP2 did not modify the ADP-induced aggregation (Fig 1A and 1B, S1 Table).

Western blot analysis of ADP-activated platelets detected an intense immunoreactive band corresponding to c-Src kinase with the same magnitude in saline- and LPS-injected rats (Fig 1C, S2 Table). In addition, phosphorylation of c-Src on tyrosine 416 residue (that indicates c-Src activation) was similar in platelets of saline- and LPS-injected rats, as showed by densitometry analysis when phosphorylated c-Src levels were normalized to total c-Src (Fig 1C, S2 Table).

### Inhibition of protein kinase C (PKC) prevents the reduced platelet aggregation of LPS-treated rats

**Fig 2:**
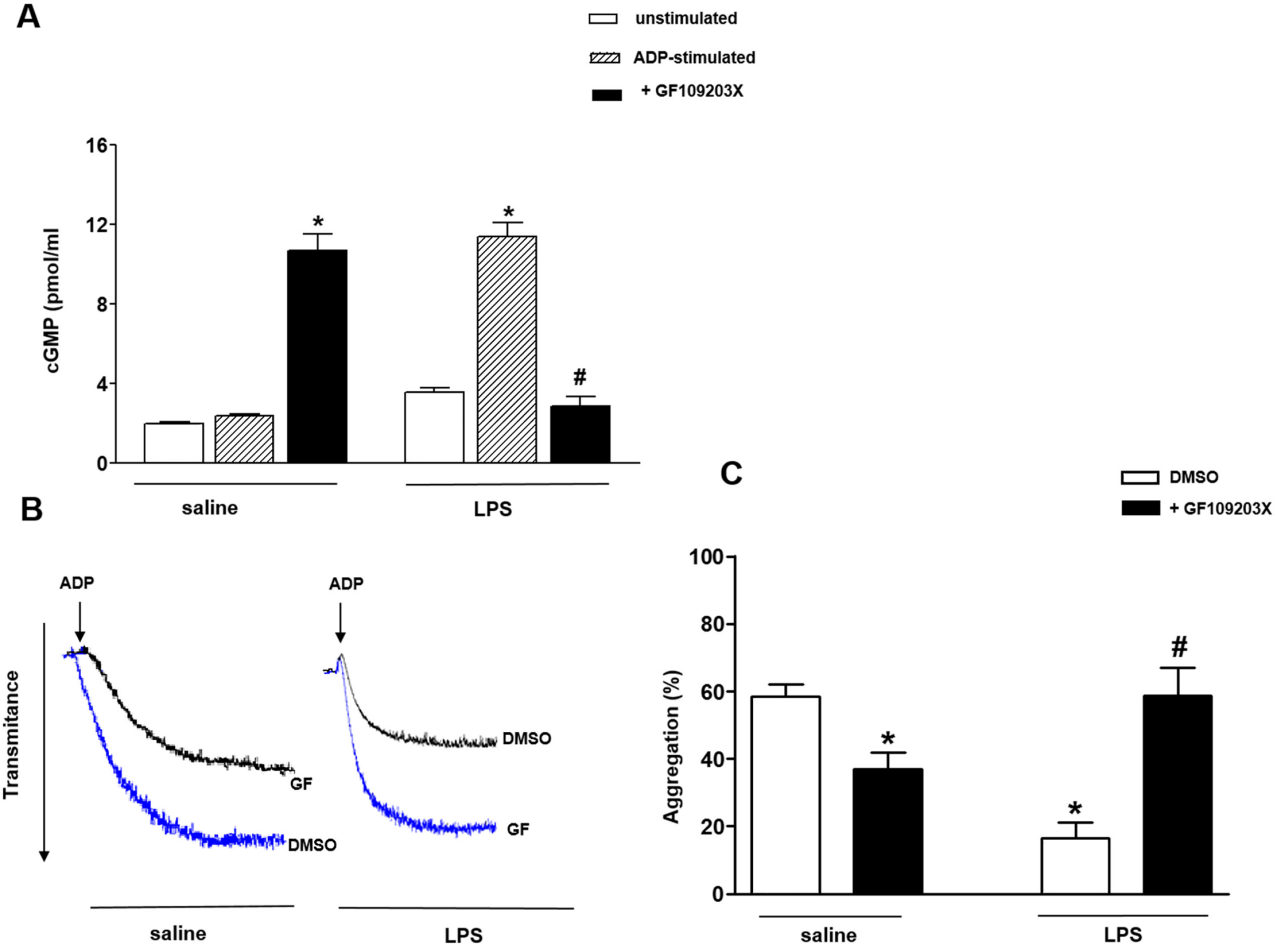
PKC inhibition decreases intraplatelet cGMP levels and increases ADP-induced platelet aggregation of LPS-treated rats. Washed platelets (1.2 x 10^8^ platelets/ml) were incubated with GF109203X (GF; 10 μM) or its vehicle (1% DMSO) for 3 min before addition of ADP (10 μM). (Panel A) Intraplatelet cGMP concentration (pmol/ml) in saline and LPS-treated rats. (Panel B) Typical aggregation curves from saline- or LPS-injected rats in absence or presence of GF109203X. (Panel C) ADP-induced platelet aggregation. Results are shown as mean ± SEM values (*n* = 4–6 different animals). *P<0.05 compared with ADP-stimulated platelets of saline-injected rats. ^#^ *P*<0.05 compared with ADP-stimulated platelets of LPS-injected rats.

In saline-injected rats, the levels of cGMP did not change significantly between non-stimulated and ADP-stimulated platelets (Fig 2A, S3 Table). In contrast, in LPS group, the cGMP levels were markedly greater in ADP-stimulated compared with non-stimulated platelets (Fig 2A, S3 Table).

In saline-injected rats, pre-incubation of ADP-stimulated platelets with the PKC inhibitor GF109203X (10 μM, 3 min) elevated by 4.5-fold the cGMP levels (Fig 2A,S3 Table) and significantly inhibited ADP-induced aggregation (Fig 2B and 2C). In LPS-treated rats, GF109203X prevented the inhibition of platelet aggregation (Fig 2B and 2C, S4 Table), which was accompanied by lower intraplatelet cGMP levels (Fig 2A).

### Inhibition of soluble guanylyl cyclase (sGC) and protein kinase G (PKG) prevents the reduced platelet aggregation of LPS-treated rats

**Fig 3:**
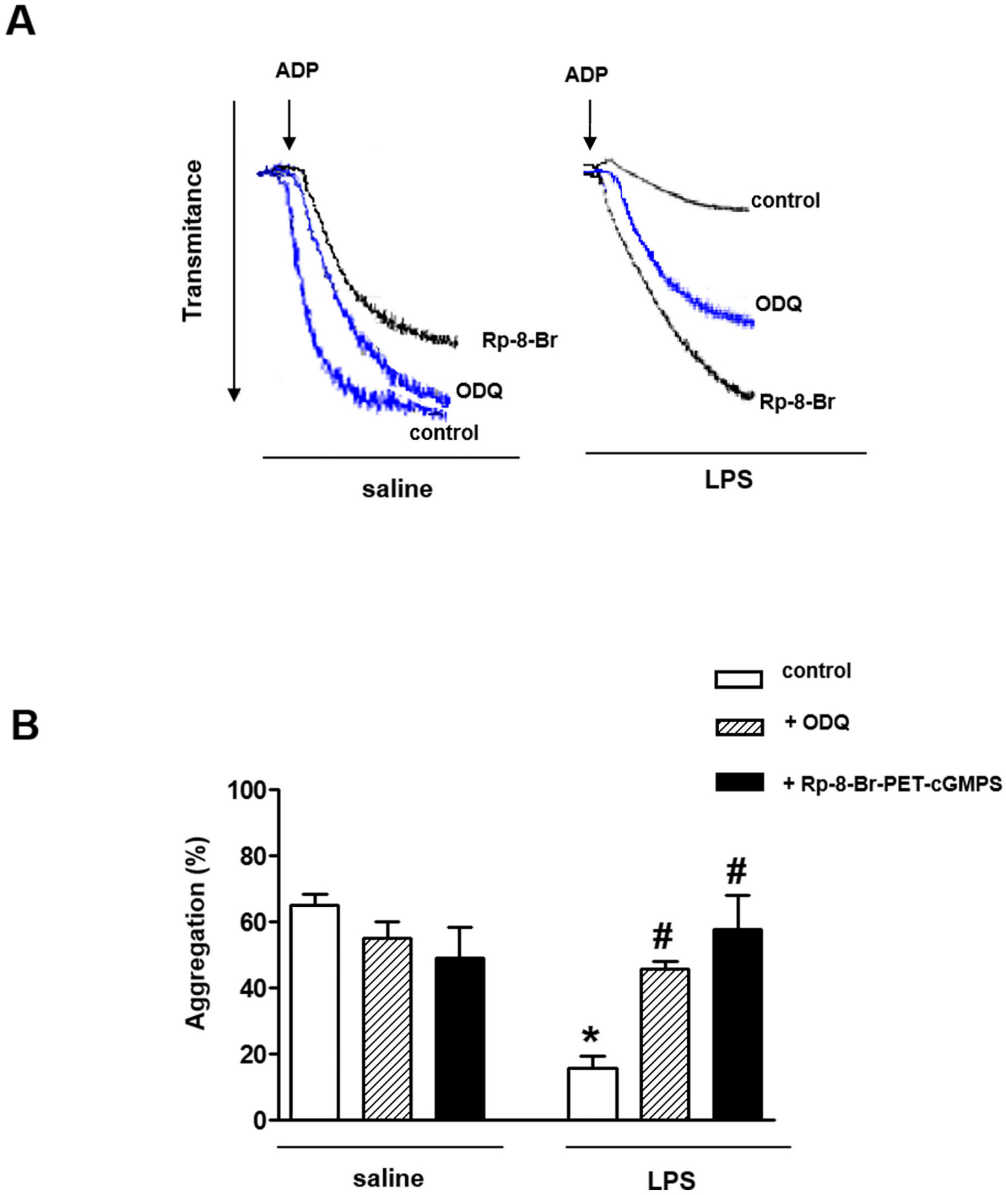
Effect of soluble guanylyl cyclase (sGC) and protein kinase G (PKG) inhibition on platelet aggregation of LPS-treated rats. (Panels A and B) Washed platelets (1.2 x 10^8^ platelets/ml) were incubated with ODQ (25 μM) or Rp-8-Br-PET-cGMPS (25 μM) for 3 min, and stimulated with ADP (10 μM). Results are shown as mean ± SEM values (*n* = 4–6 different animals). *P<0.05 compared with control values of saline-injected rats. ^#^ *P*<0.05 compared with control values of LPS-injected rats.

Prior incubation of platelets with either the sGC inhibitor ODQ (25 μM, 3 min) or the PKG inhibitor Rp-8-Br-PET-cGMPS (25 μM, 3 min) fully prevented the reduction of ADP-induced platelet aggregation by LPS (Fig 3A and 3B, S5 Table). In saline-treated rats, ODQ and Rp-8-Br-PET-cGMPS had no significant effect on platelet aggregation (Fig 3A and 3B).

### Effect of PI3K and AKT inhibition on washed platelet aggregation of LPS-treated rats

**Fig 4:**
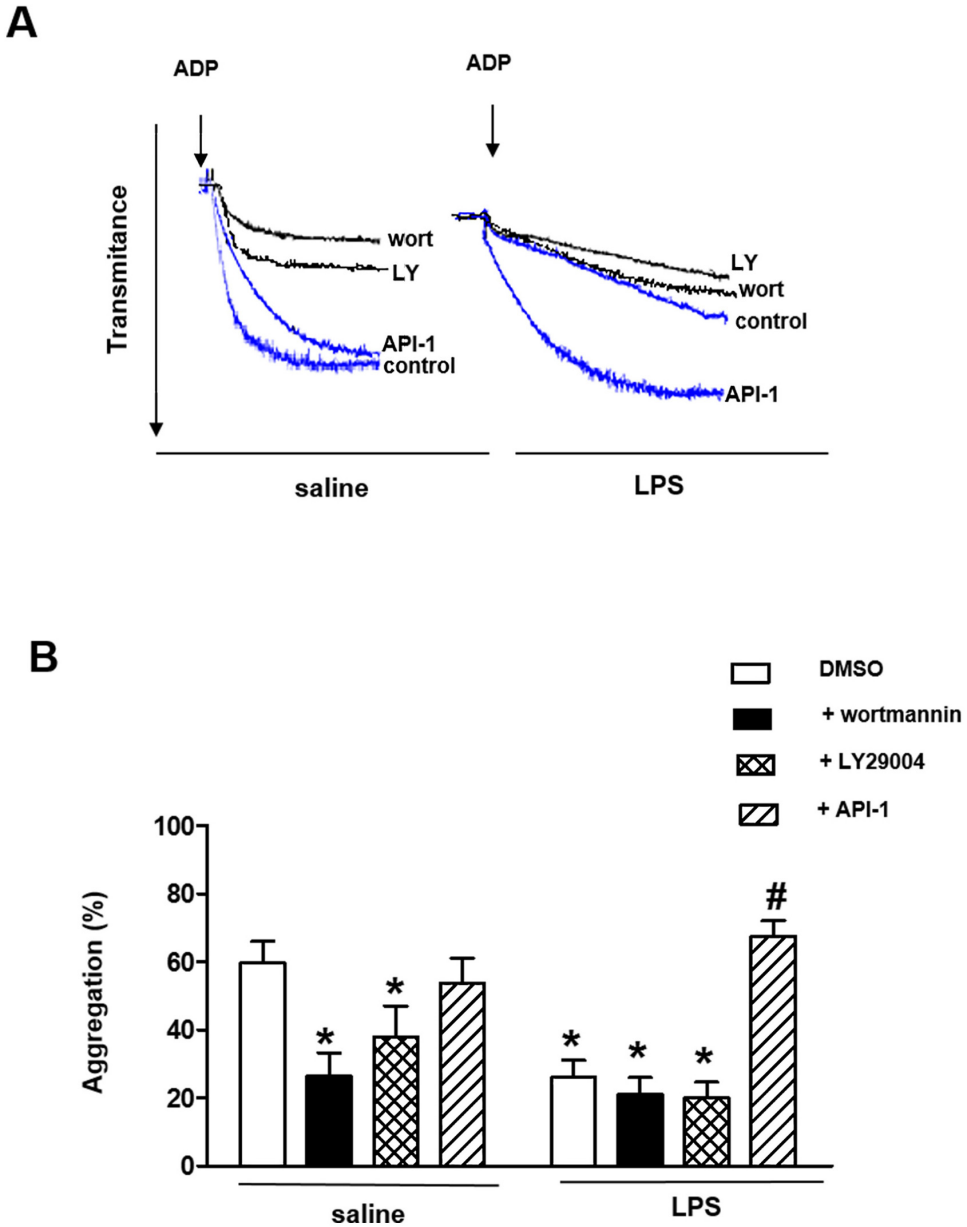
Effects of PI3K and AKT inhibitors on ADP-induced platelet aggregation. Washed platelets (1.2 x 10^8^ platelets/ml) were incubated with wortmannin (100 nM), LY29004 (10 μM), API-1 (20 μM) or its vehicle (1% DMSO) for 3 min before addition of ADP (10 μM). (Panels A) Typical aggregation curves from saline- or LPS-injected rats in absence or presence of the enzyme inhibitors. (Panel B) ADP-induced platelet aggregation. Results are shown as mean ± SEM values (*n* = 4–6 different animals). *P<0.05 compared with untreated platelets of saline-injected rats. ^#^ *P*<0.05 compared with untreated platelets of LPS-injected rats.

Prior incubation (3 min) of washed platelets from saline-injected rats with the PI3K inhibitors wortmannin (100 nM) and LY294002 (10 μM) significantly reduced ADP-induced platelet aggregation (Fig 4A and 4B, S6 Table). The AKT inhibitor API-1 (20 μM, 3 min) did not affect the platelet aggregation in saline group (Fig 4A and 4B).

In LPS-treated rats, wortmannin and LY294002 did not change the ADP-induced aggregation. However, AKT inhibition by API-1 fully prevented the inhibitory effect of LPS treatment on platelet aggregation (Fig 4A and 4B).

**Fig 5:**
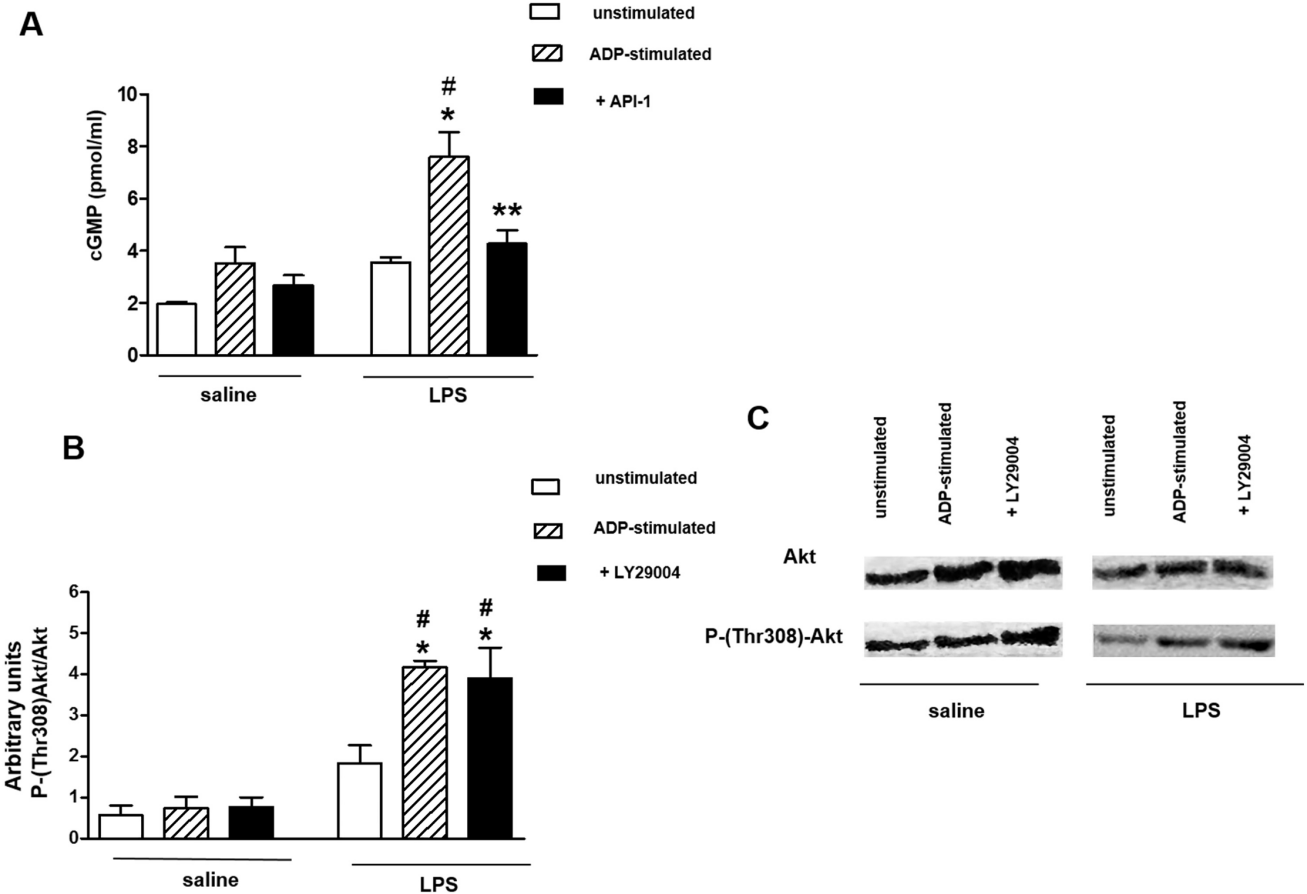
Effects of PI3K and AKT inhibitors on cGMP levels and AKT phosphorylation of LPS-injected rats. Washed platelets (1.2 x 10^8^ platelets/ml) were incubated with LY29004 (10 μM), API-1 (20 μM) or its vehicle (1% DMSO) for 3 min before addition of ADP (10 μM). (Panel A) Intraplatelet cGMP concentration (pmol/ml) in saline and LPS-treated rats. (Panels B and C) Samples (50 μg of protein/well) were loaded on SDS-PAGE 10% and analyzed by Western blot using anti-AKT or anti-phospho(Thr 308)-AKT antibodies. Graph shows densitometric analysis of immunoreactive bands. Results are shown as mean ± SEM values (*n* = 4–6 different animals). *P<0.05 compared with respective groups of saline-injected rats. **P<0.05 compared with ADP-stimulated platelets of LPS group. #*P*<0.05 compared with unstimulated platelets of LPS group.

The levels of cGMP did not change significantly between non-stimulated and ADP-stimulated platelets (treated or not with API-1). In contrast, the increases in cGMP levels in ADP-stimulated of LPS group were prevented by API-1 (Fig 5A, S7 Table).

The densitometry analysis showed that the phosphorylation of AKT in Thr308 residue was significantly higher in ADP-stimulated platelets in platelets of LPS-treated rats compared with saline group. Pre-incubation of ADP-stimulated platelets with LY29004 affected AKT phosphorylation neither in saline- nor in LPS-group (Fig 5B and 5C, S8 Table).

## Discussion

In the present work, we have demonstrated that PKC and AKT mediate the reduction of ADP-induced rat platelet aggregation after LPS-injection by upregulating upstream the sGC/cGMP/PKG pathway. In addition, we have showed that SFKs are not involved in the inhibitory effect of LPS on platelet aggregation.

Platelet activation is a complex and tightly coordinate event. Agonist binding to specific receptor initiates a cascade of intracellular signals called inside-out that is responsible for conversion of the fibrinogen receptor integrin αIIbβ3 from the low to the high affinity state [25]. The conformational change of integrin αIIbβ3 allows the ligation of fibrinogen, initiating a second activation wave known as outside-in signaling, which is important for the stabilization of platelet aggregates [5]. Activation of c-Src is a downstream event after fibrinogen binding that takes part in granule release and cytoskeletal regulation in platelets [26,27]. Previous works have demonstrated that increased cytokine production in macrophages [28, 29] and up-regulation of VCAM-1 in human tracheal smooth muscle cells [30] by LPS are mediated by Src kinase and PI3K activation. In our work, phosphorylation of c-Src at Tyr 416 residue, which indicates activation of this kinase, was of the same magnitude in platelets from control and LPS group. Besides c-Src, other members of SFKs including Fyn, Lyn, Lck and Yes have been demonstrated in platelets [31]. In physiological conditions, Lyn and Fyn negatively regulate the platelet responses by inhibiting intracellular Ca^++^ mobilization, PKC activation and integrin αIIbβ3 signaling [32,33]. In our study, the non-selective inhibition of SFKs by using PP2 did not affect the inhibition of platelet aggregation by LPS. Therefore, our results indicate that SFKs do not take part in the inhibitory effect of LPS on platelet aggregation.

Currently, the presence of NOS in platelets is controversial [34,35]. Recently, it was demonstrated that inducible NOS (iNOS) or endothelial NOS (eNOS) are present neither in mouse nor in human platelets [36]. However, various works have shown the presence of eNOS/NO/sGC/cGMP signaling in platelets using by different experimental approaches including aggregation assays in presence of sGC and eNOS inhibitors [37], western blotting for eNOS [38, 39], [^3^H]L-citrulline [40] and cGMP production [37,38]. Therefore, in the present work we decided to investigate the role of sGC/cGMP/PKG pathway in the inhibitory effect of LPS-injection on platelet aggregation. Our results showed increased levels of cGMP in ADP-activated platelets in LPS compared with saline group, indicating elevated NO formation in this condition. NO has been shown to bind to the prosthetic group containing the reduced (Fe^2+^) haem moiety in sGC, leading to the conversion of GTP to cGMP [41]. The compound ODQ inhibits sGC by oxidizing its haem moiety, thus impairing the NO binding to the enzyme [42]. Therefore, we have further used ODQ to explore the cGMP-dependent mechanisms mediating the inhibitory effect of LPS on platelet aggregation. Our results clearly show that ODQ prevents the inhibitory effect of LPS on platelet aggregation, indicating that this effect is mediated by cGMP-dependent mechanisms. PKG inhibits platelet activity through phosphorylation of IP3 receptor on dense tubular system, thus increasing Ca^++^ reuptake into the stores [10], reducing both fibrinogen receptor activation and actin polymerization [43,44]. In the present work, the PKG inhibitor Rp-8-Br-PET-cGMPS prevented the inhibition of platelet aggregation by LPS, indicating a major role for PKG activation.

Endothelial NO synthase (eNOS) is modulated by post-translational regulatory modifications, including multiple phosphorylations. The phosphorylation at Ser 1177, Ser 635 and Ser 617 stimulates eNOS activity while phosphorylations at Thr 495 and Ser 116 inhibit this enzyme activity [45]. Typically, PKC has been described as an eNOS inhibitor [46,47], but recently PKC has been shown to activate eNOS via Ser 1177 phosphorylation in endocannabinoid 2-arachidonoylglycerol-stimulated platelets [48].

The PKC family is composed of 10 members of serine/threonine protein kinases. Intracellular localization, expression and activation of PKC are dynamics processes tightly modulated by different enzymes and conditions. In the present study, incubation of platelets from control rats with the PKC inhibitor GF109203X increased the cGMP levels and inhibited aggregation, suggesting that under physiological conditions eNOS is negatively modulated by PKC. Conversely, in platelets of LPS-treated rats, GF109203X reduced the levels of cGMP and prevented the inhibition of platelet aggregation. In fact, in endothelial cells under oxidative stress conditions, δPKC activation is accompanied by eNOS stimulation via Ser 1179 phosphorylation [49]. Taken together, our results strongly suggest that different PKC isoforms positively or negatively modulate platelet activity depending on the environment condition. In platelets of LPS-injected rats, PKC increases cGMP levels, leading to inhibition of platelet aggregation through PKG-dependent mechanisms. This contrasts with healthy conditions where PKC mediates platelet activation and inhibition of cGMP production.

It is well established that AKT may activate eNOS by phosphorylation at Ser 1177 residue [46,47,50]. An increase of AKT activation through enhanced phosphorylation at Thr 318 was observed in platelets of LPS-treated rats, even in the presence of the PI3K inhibitor LY294002. Accordingly, AKT inhibition with API-1 prevented both the reduced platelet aggregation and the increased intraplatelet cGMP levels in LPS-treated rats. Nevertheless, incubation the platelets with LY294002 (or the PI3K inhibitor wortmannin) did not affect platelet aggregation in LPS group. Besides PI3K, reports have been shown that AKT may be phosphorylated by other kinases, including PKC in platelets [51] and Ca^++^/calmodulin-dependent kinases in cultured neurons [52]. Therefore, our results indicate that treatment of rats with LPS increases AKT activation in platelets via PI3K-independent pathways, which enhances eNOS activity and cGMP levels leading to inhibition of platelet aggregation.

In conclusion, our work shows that inhibition of rat platelet aggregation by LPS is mediated by cGMP/PKG-dependent mechanisms. In addition, PKC and AKT take place in the inhibitory effects of LPS in platelet aggregation by acting as upstream modulators of cGMP/PKG pathway.

## Supporting Information

S1 Table Data of platelet aggregation of rats treated with saline or LPS (6 h). Platelets were incubated with 1% DMSO (vehicle) or the Src inhibitor PP2 (10 μM) for 3 min before ADP (10 μM) addition. Values are presented as means ± S.E.M. (n = 4 different animals in each group)
(PDF)

S2 Table Ratio of densitometric values of immunoreactive band of phosphorylated (P-Tyr416) and non-phosphorylate forms of Src in platelets of rats treated with saline or LPS (6 h). Washed platelets were stimulated or not with ADP (10 μM). Values are presented as means ± S.E.M. (n = 3 different animals in each group)
(PDF)

S3 Table Data of values of intraplatelet cGMP levels of rats treated with saline or LPS (6 h). Platelets were incubated with the non-selective PKC inhibitor GF109203X (10 μM) or 1% DMSO (vehicle) for 3 min prior addition of or ADP (10 μM). Values are presented as means ± S.E.M. (n = 4–6 different animals in each group)
(PDF)

S4 Table Data of platelet aggregation of rats treated with saline or LPS (6 h). Platelets were incubated with 1% DMSO (vehicle) or the PKC inhibitor GF109203X (10 μM) for 3 min before ADP (10 μM) addition. Values are presented as means ± S.E.M. (n = 4–6 different animals in each group)
(PDF)

S5 Table Data of platelet aggregation of rats injected with saline or LPS (6 h). Platelets were incubated or not with either the soluble guanylyl cyclase inhibitor ODQ (25 μM) or the protein kinase G inhibitor Rp-8-Br-PET-cGMPS (25 μM) for 3 min before ADP (10 μM) addition. Values are presented as means ± S.E.M. (n = 4–6 different animals in each group)
(PDF)

S6 Table Data of platelet aggregation of rats injected with saline or LPS (6 h). Platelets were incubated with wortmannin (100 nM, PI3K inhibitor), LY29004 (10 μM, PI3K inhibitor), API-1 (20 μM, AKT inhibitor) or 1% DMSO (vehicle) for 3 min before ADP (10 μM) addition. Values are presented as means ± S.E.M. (n = 4–6 different animals in in each group)
(PDF)

S7 Table Data of intraplatelet cGMP levels of rats treated with saline or LPS (6 h). Platelets were incubated with the AKT inhibitor API-1 (20 μM) for 3 min prior to addition of ADP (10 μM). Values are presented as means ± S.E.M. (n = 4–6 different animals in each group)
(PDF)

S8 Table Ratio of densitometric values of immunoreactive band of phosphorylated (P-Thr308) and non-phosphorylate forms of AKT in platelets of rats treated with saline or LPS (6 h). Platelets were incubated with the PI3K inhibitor LY29004 (10 μM) or its vehicle DMSO (1%) for 3 min prior addition of ADP (10 μM). Values are presented as means ± S.E.M. (n = 3 different animals in each group)
(PDF)
