# Supplementary material for: PKC and AKT Modulate cGMP/PKG Signaling Pathway on Platelet Aggregation in Experimental Sepsis
Source: PLoS One. 2015 Sep 16;10(9):e0137901. doi: 10.1371/journal.pone.0137901 (PMC4573322; doi:10.1371/journal.pone.0137901)
Supplement: S1 Table — Platelets were incubated with 1% DMSO (vehicle) or the Src inhibitor PP2 (10 μM) for 3 min before ADP (10 μM) addition. Values are presented as means ± S.E.M. (n = 4 different animals in each group) (PDF) [file pone.0137901.s001.pdf]

S1 table Data of platelet aggregation of rats treated with saline or LPS (6 h). Platelets were incubated with 1% DMSO (vehicle) or the Src inhibitor PP2 (10 μM) for 3 min before ADP (10 μM) addition. Values are presented as means ± S.E.M. (n= 4 different animals in each group).

	**Saline group**		**LPS group**	
	**Mean**	**S.E.M**	**Mean**	**S.E.M**
**Platelet + DMSO**	**72**	**3.0**	**26.0**	**5.0**
**Platelet + PP2**	**53.7**	**7.0**	**38**	**3.0**
