# Supplementary material for: PKC and AKT Modulate cGMP/PKG Signaling Pathway on Platelet Aggregation in Experimental Sepsis
Source: PLoS One. 2015 Sep 16;10(9):e0137901. doi: 10.1371/journal.pone.0137901 (PMC4573322; doi:10.1371/journal.pone.0137901)
Supplement: S2 Table — Washed platelets were stimulated or not with ADP (10 μM). Values are presented as means ± S.E.M. (n = 3 different animals in each group) (PDF) [file pone.0137901.s002.pdf]

S2 table Ratio of densitometric values of immunoreactive band of phosphorylated (P-Tyr416) and non-phosphorylate forms of Src in platelets of rats treated with saline or LPS (6 h). Washed platelets were stimulated or not with ADP (10 µM). Values are presented as means ± S.E.M. (n= 3 different animals in each group)

	**Saline group**		**LPS group**	
	MEAN	***S.E.M.***	***MEAN***	***S.E.M.***
**Platelet**	**2.1**	**0.2**	**1.9**	**0.4**
**Platelet + ADP**	**2.0**	**0.6**	**1.8**	**0.2**
