# Supplementary material for: PKC and AKT Modulate cGMP/PKG Signaling Pathway on Platelet Aggregation in Experimental Sepsis
Source: PLoS One. 2015 Sep 16;10(9):e0137901. doi: 10.1371/journal.pone.0137901 (PMC4573322; doi:10.1371/journal.pone.0137901)
Supplement: S3 Table — Platelets were incubated with the non-selective PKC inhibitor GF109203X (10 μM) or 1% DMSO (vehicle) for 3 min prior addition of or ADP (10 μM). Values are presented as means ± S.E.M. (n = 4–6 different animals in each group) (PDF) [file pone.0137901.s003.pdf]

S3 table Data of values of intraplatelet cGMP levels of rats treated with saline or LPS (6 h). Platelets were incubated with the non-selective PKC inhibitor GF109203X (10 μM) or 1% DMSO (vehicle) for 3 min prior addition of or ADP (10 μM). Values are presented as means ± S.E.M. (n= 4-6 different animals in each group).

	**Saline group**		**LPS group**	
	MEAN	***S.E.M.***	***MEAN***	***S.E.M.***
**Platelet**	**1.9**	**0.1**	**3.5**	**0.2**
**Platelet + ADP**	**2.4**	**0.1**	**11.4**	**0.8**
**Platelet + GF109203X + ADP**	**10.7**	**0.9**	**2.8**	**0.5**
