# Supplementary material for: PKC and AKT Modulate cGMP/PKG Signaling Pathway on Platelet Aggregation in Experimental Sepsis
Source: PLoS One. 2015 Sep 16;10(9):e0137901. doi: 10.1371/journal.pone.0137901 (PMC4573322; doi:10.1371/journal.pone.0137901)
Supplement: S4 Table — Platelets were incubated with 1% DMSO (vehicle) or the PKC inhibitor GF109203X (10 μM) for 3 min before ADP (10 μM) addition. Values are presented as means ± S.E.M. (n = 4–6 different animals in each group) (PDF) [file pone.0137901.s004.pdf]

S4 table Data of platelet aggregation of rats treated with saline or LPS (6 h). Platelets were incubated with 1% DMSO (vehicle) or the PKC inhibitor GF109203X (10 μM) for 3 min before ADP (10 μM) addition. Values are presented as means ± S.E.M. (n= 4-6 different animals in each group)

	**Saline group**		**LPS group**	
	MEAN	*S.E.M.*	*MEAN*	*S.E.M.*
**Platelet + DMSO**	**56.9**	**3.0**	**15.4**	**4.6**
**Platelet + GF109203X**	**36.9**	**4.6**	**58.5**	**7.7**
