# Supplementary material for: PKC and AKT Modulate cGMP/PKG Signaling Pathway on Platelet Aggregation in Experimental Sepsis
Source: PLoS One. 2015 Sep 16;10(9):e0137901. doi: 10.1371/journal.pone.0137901 (PMC4573322; doi:10.1371/journal.pone.0137901)
Supplement: S5 Table — Platelets were incubated or not with either the soluble guanylyl cyclase inhibitor ODQ (25 μM) or the protein kinase G inhibitor Rp-8-Br-PET-cGMPS (25 μM) for 3 min before ADP (10 μM) addition. Values are presented as means ± S.E.M. (n = 4–6 different animals in each group) (PDF) [file pone.0137901.s005.pdf]

S5 table Data of platelet aggregation of rats injected with saline or LPS (6 h). Platelets were incubated or not with either the soluble guanylyl cyclase inhibitor ODQ (25 μM) or the protein kinase G inhibitor Rp-8-Br-PET-cGMPS (25 μM) for 3 min before ADP (10 μM) addition. Values are presented as means ± S.E.M. (n = 4-6 different animals in each group).

	**Saline group**		**LPS group**	
	MEAN	*S.E.M.*	*MEAN*	*S.E.M.*
**Platelet + ADP**	**66.0**	**2.0**	**16.0**	**3.0**
**Platelet + ODQ + ADP**	**56.0**	**4.0**	**46.0**	**2.0**
**Platelet + Rp-8-Br-PET-cGMPS + ADP**	**50.0**	**9.0**	**58.0**	**10.0**
