# Supplementary material for: PKC and AKT Modulate cGMP/PKG Signaling Pathway on Platelet Aggregation in Experimental Sepsis
Source: PLoS One. 2015 Sep 16;10(9):e0137901. doi: 10.1371/journal.pone.0137901 (PMC4573322; doi:10.1371/journal.pone.0137901)
Supplement: S6 Table — Platelets were incubated with wortmannin (100 nM, PI3K inhibitor), LY29004 (10 μM, PI3K inhibitor), API-1 (20 μM, AKT inhibitor) or 1% DMSO (vehicle) for 3 min before ADP (10 μM) addition. Values are presented as means ± S.E.M. (n = 4–6 different animals in in each group) (PDF) [file pone.0137901.s006.pdf]

S6 table Data of platelet aggregation of rats injected with saline or LPS (6 h). Platelets were incubated with wortmannin (100 nM, PI3K inhibitor), LY29004 (10 µM, PI3K inhibitor), API-1 (20 µM, AKT inhibitor) or 1% DMSO (vehicle) for 3 min before ADP (10 µM) addition. Values are presented as means ± S.E.M. (n= 4-6 different animals in each group).

	**Saline group**		**LPS group**	
	MEAN	*S.E.M.*	*MEAN*	*S.E.M.*
**Platelet + DMSO**	**60.0**	**6.0**	**26.0**	**4.0**
**Platelet + wortmannin**	**28.0**	**6.0**	**22.0**	**4.0**
**Platelet + LY29004**	**38.0**	**8.0**	**20.0**	**4.0**
**Platelet + API-1**	**54.0**	**8.0**	**68.0**	**4.0**
