# Supplementary material for: PKC and AKT Modulate cGMP/PKG Signaling Pathway on Platelet Aggregation in Experimental Sepsis
Source: PLoS One. 2015 Sep 16;10(9):e0137901. doi: 10.1371/journal.pone.0137901 (PMC4573322; doi:10.1371/journal.pone.0137901)
Supplement: S7 Table — Platelets were incubated with the AKT inhibitor API-1 (20 μM) for 3 min prior to addition of ADP (10 μM). Values are presented as means ± S.E.M. (n = 4–6 different animals in each group) (PDF) [file pone.0137901.s007.pdf]

S7 table Data of intraplatelet cGMP levels of rats treated with saline or LPS (6 h). Platelets were incubated with the AKT inhibitor API-1 (20 μM) for 3 min prior to addition of ADP (10 μM). Values are presented as means ± S.E.M. (n= 4-6 different animals in each group).

	**Saline group**		**LPS group**	
	MEAN	*S.E.M.*	*MEAN*	*S.E.M.*
**Platelet**	**2.0**	**0.1**	**3.6**	**0.2**
**Platelet + ADP**	**3.6**	**0.5**	**7.6**	**1.0**
**Platelet + API-1 + ADP**	**2.6**	**0.4**	**4.2**	**0.4**
