# Supplementary material for: PKC and AKT Modulate cGMP/PKG Signaling Pathway on Platelet Aggregation in Experimental Sepsis
Source: PLoS One. 2015 Sep 16;10(9):e0137901. doi: 10.1371/journal.pone.0137901 (PMC4573322; doi:10.1371/journal.pone.0137901)
Supplement: S8 Table — Platelets were incubated with the PI3K inhibitor LY29004 (10 μM) or its vehicle DMSO (1%) for 3 min prior addition of ADP (10 μM). Values are presented as means ± S.E.M. (n = 3 different animals in each group) (PDF) [file pone.0137901.s008.pdf]

S8 table Ratio of densitometric values of immunoreactive band of phosphorylated (P-Thr308) and non-phosphorylate forms of AKT in platelets of rats treated with saline or LPS (6 h). Platelets were incubated with the PI3K inhibitor LY29004 (10 µM) or its vehicle DMSO (1%) for 3 min prior addition of ADP (10 µM). Values are presented as means ± S.E.M. (n= 3 different animals in each group)

	**Saline group**		**LPS group**	
	MEAN	*S.E.M.*	*MEAN*	*S.E.M.*
**Platelet**	**0.5**	**0.2**	**1.8**	**0.4**
**Platelet + ADP**	**0.8**	**0.3**	**4.1**	**0.1**
**Platelet + LY29004 + ADP**	**0.8**	**0.2**	**3.9**	**0.6**
